# ECAB-SegFormer: A Boundary-Aware and Efficient Channel Attention Network for *Ulva prolifera* Semantic Segmentation in Remote Sensing Imagery

**DOI:** 10.3390/s26072166

**Published:** 2026-03-31

**Authors:** Yue Liang, Danyang Cao, Zice Ji, Hao Yang, Maohua Guo, Xiaoya Liu, Xutong Guo, Jiahao Wu, Yulong Song, Shanzhe Zhang

**Affiliations:** 1 School of Artificial Intelligence and Computer, North China University of Technology, Beijing 100144, China; 23101020105@mail.ncut.edu.cn (Y.L.); 23101020110@mail.ncut.edu.cn (Z.J.); liuxiaoya5992@163.com (X.L.); guoxutong040928@163.com (X.G.); 23101020125@mail.ncut.edu.cn (J.W.); 2 School of Computer and Artificial Intelligence, Beijing Technology and Business University, Beijing 100048, China; pikx258@163.com (H.Y.); sungyulung@163.com (Y.S.); zhangsz@btbu.edu.cn (S.Z.); 3 National Satellite Ocean Application Service, Beijing 100081, China; gmh@mail.nsoas.org.cn; 4 Key Laboratory of Space Ocean Remote Sensing and Application, Ministry of Natural Resources, Beijing 100081, China

**Keywords:** SegFormer, *Ulva prolifera*, ECA channel attention, boundary supervision, remote sensing imagery, semantic segmentation

## Abstract

**Highlights:**

**What are the main findings?**

- An optimized SegFormer architecture tailored for *Ulva prolifera* semantic segmentation is developed by integrating a boundary supervision mechanism in parallel with the decoder. The boundary branch generates prediction maps from shallow features and is guided by supervision signals derived via a morphological gradient, enabling explicit boundary learning. This design significantly improves contour sensitivity and fitting accuracy while reducing missed detections and boundary discontinuities in complex backgrounds.
- To further enhance feature representation, an Efficient Channel Attention (ECA) module is embedded between shallow encoder features and the boundary supervision branch to perform adaptive channel-wise reweighting. This module strengthens informative features while suppressing background noise from marine and atmospheric conditions with negligible computational overhead. Experimental results demonstrate that the proposed method achieves superior boundary segmentation accuracy and maintains strong model lightweightness compared to mainstream approaches.

**What is the implication of the main finding?**

- The proposed method, which integrates the boundary supervision mechanism with the ECA module, significantly improves the accuracy and reliability of *Ulva prolifera* semantic segmentation. It enables more precise characterization of spatial distribution and dynamic changes, which is crucial for marine ecological protection, as *Ulva prolifera* blooms can substantially impact marine ecosystems, fisheries, coastal tourism, and maritime transportation.
- Built upon an optimized SegFormer framework, the proposed approach provides an efficient and practical solution for large-scale algal bloom monitoring, with potential for real-time deployment. It effectively addresses challenges such as boundary blurring, background noise, and complex marine environments, thereby promoting the application of deep learning techniques in remote sensing-based marine ecological monitoring and supporting timely warning, response, and coastal management decisions.

**Abstract:**

To achieve high-precision *Ulva prolifera* semantic segmentation from remote sensing imagery and address issues such as boundary fragmentation, contour dilation, and missed segmentation of scattered patches under complex marine backgrounds, this paper proposes an improved SegFormer-based network termed ECAB-SegFormer. The proposed method enhances near-infrared feature representation and boundary perception by embedding an Efficient Channel Attention (ECA) module into shallow features and introducing a boundary supervision branch. Experimental results on the HYU dataset demonstrate that the proposed method achieves consistent improvements over classical baseline models and further outperforms several representative modern strong segmentation baselines. Compared with advanced methods such as **DeepLabV3+**, **Swin-Unet**, and **Gated-SCNN**, the proposed model achieves maximum improvements of **2.77%**, **5.80%**, and **4.26(pixel)** in **mIoU**, **BFScore**, and **Hausdorff Distance (HD)**, respectively, while also obtaining superior **Precision** and **F1 Scores**. These results demonstrate significant advantages in both regional segmentation accuracy and boundary localization quality, validating the effectiveness, robustness, and practical potential of the proposed method for *Ulva prolifera* semantic segmentation in remote sensing applications.

## 1. Introduction

*Ulva prolifera* (green tide) is one of the largest floating algal bloom phenomena worldwide, with major outbreaks occurring in the Yellow Sea, causing severe impacts on the marine ecological environment, fisheries, coastal tourism, and maritime transportation [1,2]. Therefore, rapid and accurate monitoring of its spatial distribution and dynamics using satellite remote sensing is of great significance for marine ecological security and coastal economic development [3].

However, due to spectral confusion with underlying seawater, suspended matter, turbidity variations, and sea surface foam, accurately extracting scattered *Ulva prolifera* patches from remote sensing imagery remains challenging [4]. In addition, its floating morphology is strongly affected by winds and currents, resulting in irregular and dynamic boundaries that often lead to false negatives and false positives during segmentation [5].

Traditional remote sensing approaches for *Ulva prolifera* monitoring primarily rely on empirical spectral indices, such as the Floating Algae Index (FAI) and Normalized Difference Phytoplankton Index (NDPI), which can enhance the contrast between algae and water bodies to some extent [6,7]. Nevertheless, due to complex sea states and spectral variability under different imaging conditions, these index-based methods often suffer from threshold sensitivity and limited generalization, making them inadequate for large-scale, real-time, and high-precision monitoring [8].

With the rapid development of deep learning, convolutional neural networks (CNNs) have demonstrated strong feature extraction capabilities in remote sensing image analysis [9]. Deep learning methods have been widely applied to *Ulva prolifera* semantic segmentation and have significantly outperformed traditional approaches under complex marine backgrounds [10].

Fully Convolutional Networks (FCNs) enable end-to-end pixel-level prediction and have proven effective for large-scale *Ulva prolifera* extraction [11]. U-Net improves boundary integrity through encoder–decoder skip connections [12,13], while High-Resolution Networks (HRNet) preserve fine-grained details via parallel multi-scale feature processing [14]. PSPNet and DeepLab further enhance boundary delineation by aggregating multi-scale contextual information [15,16]. More recently, SegFormer, with its lightweight Transformer architecture and efficient MLP decoder, has further improved the robustness and accuracy of algal bloom semantic segmentation [17].

In addition to generic semantic segmentation frameworks, boundary-aware segmentation methods have attracted increasing attention in fine-grained target extraction tasks [18]. Existing studies typically enhance contour representation by introducing explicit edge branches, auxiliary boundary supervision losses, or joint semantic-boundary learning strategies [19]. For example, some approaches employ dedicated boundary branches to explicitly model object contours, thereby improving contour continuity and boundary integrity [20], while others adopt multi-task learning frameworks to jointly optimize region segmentation and boundary prediction, alleviating boundary ambiguity and fragmented segmentation results [21]. These methods have demonstrated strong boundary recovery capabilities in both natural image segmentation and high-precision remote sensing applications [22].

However, most existing boundary-aware methods are primarily designed for generic objects or terrestrial remote sensing scenarios [23], with limited consideration of the low-contrast boundaries, irregular floating morphology, and complex background interference characteristics of *Ulva prolifera* in marine remote sensing imagery. In particular, when small scattered patches and large continuous boundaries coexist, conventional boundary supervision strategies often struggle to balance local detail recovery and global semantic consistency [24]. Therefore, designing a lightweight architecture with stronger boundary sensitivity and targeted feature enhancement remains of significant research value for *Ulva prolifera* semantic segmentation.

Despite these advances, existing feature extraction networks still face limitations in complex marine environments. On the one hand, environmental noise and background interference from clouds, waves, and reefs restrict the ability to identify scattered algal patches, leading to missed detections. On the other hand, in low-contrast regions, boundary contours of *Ulva prolifera* are often incompletely captured, resulting in segmentation gaps. Furthermore, most current methods directly adopt generic semantic segmentation models for remote sensing without targeted adaptations for the unique characteristics of *Ulva prolifera*, thereby constraining their practical applicability.

This study aims to address the limited applicability of existing models for *Ulva prolifera* monitoring in the Yellow Sea by proposing an enhanced SegFormer-based semantic segmentation framework optimized for complex marine conditions.

### Contributions

The main contributions of this work, highlighting the methodological innovations and their distinctions from existing studies, are summarized as follows:

- Unlike existing generic boundary-aware segmentation methods that are primarily designed for natural images or terrestrial remote sensing targets, we introduce a dedicated boundary supervision branch specifically tailored to the low-contrast and irregular boundaries of floating *Ulva prolifera*, which improves contour continuity and fine-structure preservation.
- To further enhance feature discrimination in shallow layers, lightweight ECA channel attention modules are embedded into $C_{1}$ and $C_{2}$. This design strengthens edge-sensitive and texture-related feature representation with minimal computational overhead, making it particularly suitable for small scattered patches and complex marine backgrounds.
- Extensive experiments and comparative analyses against classical CNN-based models, recent Transformer-based architectures, and representative boundary-aware segmentation methods demonstrate that the proposed framework achieves superior performance in both global semantic consistency and local boundary accuracy.

## 2. Related Work

Traditional methods for *Ulva prolifera* semantic segmentation mainly rely on empirically designed spectral indices, such as the Floating Algae Index (FAI) and the Normalized Difference Phytoplankton Index (NDPI). To improve robustness under marine environmental interference, researchers have proposed enhanced indices and adaptive threshold strategies. For instance, Cao et al. [25] proposed the Algal Bloom Detection Index (ABDI), while Hu [26] introduced FAI and an atmospheric correction strategy to improve cross-scene applicability. Other studies, including regional chlorophyll-based monitoring [27] and water-specific spectral indices [28], further improved detection capability in particular environments. Although these methods are computationally efficient, they remain highly dependent on threshold settings and are sensitive to suspended matter and atmospheric variations, which limits their robustness in large-scale and precise segmentation tasks.

With the development of deep learning, CNN-based methods have been increasingly applied to *Ulva prolifera* analysis. Early studies mainly focused on object detection frameworks [29], including FRCNN, RFCN, SSD [30], lightweight YOLO variants [31], and improved Cascade R-CNN models [32]. These methods improved deployment efficiency and inference speed compared with traditional spectral index approaches. However, object detection methods cannot provide pixel-level boundaries, making them insufficient for accurate distribution mapping and fine-grained ecological monitoring.

Semantic segmentation methods [33,34] further advanced pixel-level extraction of *Ulva prolifera*. Representative methods include Mask R-CNN [35,36], which was introduced into algal bloom monitoring by Jesus et al. [37], and CNN-based comparative studies with different backbones [38]. More recently, several specialized architectures have been developed, including attention-enhanced U-Net variants [39,40], synthetic-data-assisted segmentation frameworks [41,42], and Transformer-based hybrid models such as HySwinFormer [43]. These methods have significantly improved segmentation accuracy, yet challenges remain in balancing computational efficiency, boundary precision, and robustness under complex marine interference.

Recently, Transformer-based segmentation frameworks have attracted increasing attention due to their strong global modeling capability. Among them, SegFormer has achieved a favorable balance between accuracy and efficiency through its lightweight encoder and all-MLP decoder. Existing improvements mainly focus on integrating channel attention modules, such as ECA [44,45], or combining ASPP and advanced upsampling mechanisms [46,47]. Although these methods improve contextual modeling and feature recovery, they still lack targeted optimization for the low-contrast boundaries, irregular morphology, and complex spectral background of *Ulva prolifera* in marine remote sensing imagery.

Boundary-aware segmentation methods have attracted increasing attention in recent years for addressing common issues in semantic segmentation tasks, such as blurred boundaries, contour discontinuity, and loss of fine-grained structures [48]. Existing studies typically enhance contour modeling by introducing explicit boundary branches, boundary supervision loss functions, or joint region-boundary optimization frameworks [18,19,20,21,22,23,24]. A representative category of methods employs dedicated edge branches to explicitly learn object contours, where auxiliary supervision guides the backbone network to focus on high-frequency boundary details, thereby improving contour continuity and local structural integrity [49]. For example, Gated-SCNN introduces an additional shape stream parallel to the semantic branch and utilizes a gating mechanism to dynamically fuse semantic and boundary features, achieving strong boundary recovery performance in natural image segmentation tasks [18]. In addition, several studies further improve boundary localization accuracy by incorporating boundary-constrained optimization objectives, such as Boundary Loss, Dice Loss, or Hausdorff Distance Loss [50].

In remote sensing image segmentation, boundary-aware strategies have also been widely applied to high-precision tasks such as building extraction, road segmentation, and coastline detection [51,52,53]. Due to the large-scale variation in remote sensing targets, complex backgrounds, and boundary sensitivity to noise interference, region-level supervision alone is often insufficient to constrain contour information, making explicit boundary supervision particularly effective for improving object completeness and edge continuity [54]. However, most existing boundary-aware methods are primarily designed for terrestrial targets, such as buildings, roads, and agricultural parcels, whose boundaries generally exhibit relatively regular geometric structures [55]. In contrast, floating *Ulva prolifera* in marine remote sensing imagery presents substantially different characteristics, including highly irregular contours, fragmented local morphology, dense small-scale patches, and low-contrast boundaries against seawater backgrounds [56]. These characteristics make conventional boundary branch methods prone to over-smoothing, local contour discontinuity, and missed segmentation of scattered regions [57].

Compared with existing representative boundary-aware methods, the proposed approach differs significantly in design philosophy. First, instead of adopting complex dual-stream architectures or independent shape flows, such as those used in Gated-SCNN, this study preserves the original lightweight SegFormer backbone and introduces only an auxiliary boundary supervision branch at the decoder stage, thereby enhancing boundary sensitivity with minimal parameter overhead. Second, the boundary labels are not obtained through additional manual annotation but are automatically generated from semantic masks via morphological gradient operations, which reduces annotation cost and improves method transferability. More importantly, the proposed framework further integrates shallow-layer ECA channel attention to strengthen near-infrared-sensitive features around boundary regions, enabling boundary supervision to improve not only spatial contour recovery but also spectral discriminability in low-contrast regions. This collaborative design of ”shallow feature enhancement + lightweight boundary supervision” allows the proposed method to better balance global semantic consistency and local boundary integrity under complex marine backgrounds.

## 3. Improved SegFormer-Based *Ulva prolifera* Semantic Segmentation Network

### 3.1. SegFormer

SegFormer is a semantic segmentation network proposed by researchers from NVIDIA and the University of Hong Kong in 2021. It combines the advantages of Transformer-based architectures and convolutional neural networks, enabling strong semantic representation while maintaining high computational efficiency. The main characteristics of SegFormer can be summarized as follows:

1. **Lightweight MLP decoder**
  The decoder adopts a simple multilayer perceptron (MLP) structure to efficiently fuse multi-scale features from different encoder stages. This design significantly reduces computational complexity while maintaining competitive segmentation performance.
2. **Hierarchical Transformer encoder**.
  SegFormer employs a hierarchical Transformer encoder to extract multi-scale features. Unlike traditional Transformer architectures, it does not rely on positional encoding, which improves its flexibility and robustness when processing images with varying resolutions.
3. **Simple and robust architecture**.
  The overall network architecture is streamlined and parameter-efficient, enabling SegFormer to achieve a favorable balance between accuracy and inference speed. This makes it well suited for a wide range of semantic segmentation tasks, including remote sensing image analysis.

Within the family of Transformer-based networks, SegFormer emphasizes both robustness and effectiveness. It demonstrates resilience to image perturbations while achieving efficient and accurate semantic segmentation. Building upon SegFormer, further improvements can be made to address the challenges of *Ulva prolifera* semantic segmentation in remote sensing imagery.

### 3.2. Improvement Strategy

**Figure 1:**
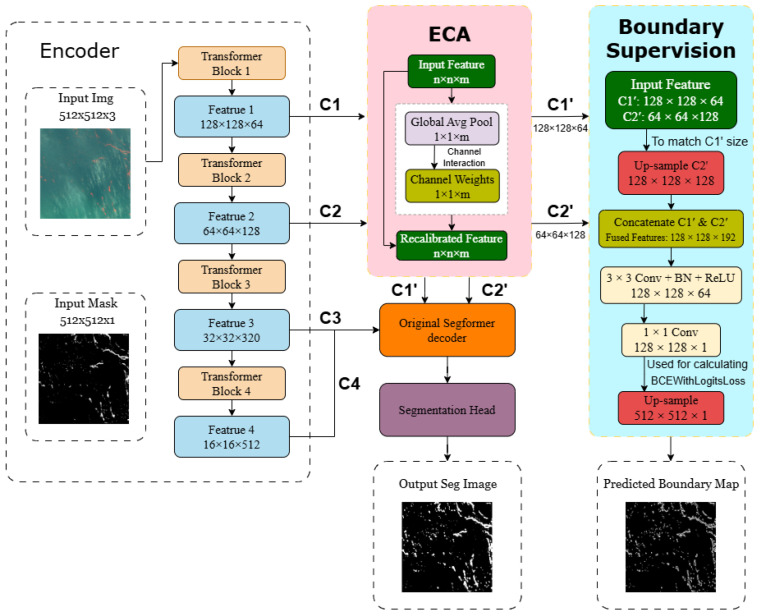
Overall architecture of the proposed improved network.

The overall architecture of the proposed improved network is illustrated in Figure 1, which highlights two main enhancements:

1. **Efficient Channel Attention (ECA) integration.**
  To address the insufficient feature representation capability of SegFormer in extracting *Ulva prolifera* from remote sensing imagery, an Efficient Channel Attention (ECA) module is introduced to enhance channel-wise feature representation. The module replaces conventional fully connected operations with a lightweight one-dimensional convolution, enabling efficient inter-channel information interaction while avoiding significant parameter overhead. It is embedded between the encoder and decoder of SegFormer, taking shallow features $C_{1}$ and $C_{2}$ as inputs and producing recalibrated features $C_{1}^{\prime }$ and $C_{2}^{\prime }$. This process strengthens the representation of near-infrared (NIR) channel information. Specifically, the ECA module employs a local cross-channel interaction mechanism to adaptively model channel-wise dependencies, allowing the network to emphasize informative feature responses while suppressing irrelevant background noise, thereby improving the discriminability of *Ulva prolifera* in complex marine environments.
2. **Boundary supervision branch.**
  To alleviate boundary ambiguity in *Ulva prolifera* segmentation, a boundary supervision branch is designed in parallel with the main decoder. This branch takes the ECA-enhanced shallow features $C_{1}^{\prime }$ and $C_{2}^{\prime }$ as inputs to generate boundary prediction maps. Meanwhile, ground-truth boundary labels are extracted from the original mask images using a morphological gradient operation. The predicted boundary maps are then supervised by an edge-aware loss function (BCEWithLogitsLoss), which guides the training process to explicitly learn boundary information. This mechanism effectively strengthens boundary feature representation during training, ensuring that edge details are preserved and enhanced in the final predictions. Consequently, the proposed method not only achieves accurate segmentation of algal regions but also precisely delineates their irregular contours, significantly reducing boundary errors caused by sparse distributions and complex morphologies.

#### 3.2.1. Efficient Channel Attention (ECA) Module

The shallow features of the SegFormer encoder ($C_{1}:128\times 128\times 64$, $C_{2}:64\times 64\times 128$) retain rich edge and texture information; however, they are also susceptible to noise and background interference. To enhance the discriminability of these shallow representations without modifying the backbone or the main decoder structure, an **Efficient Channel Attention (ECA)** module is introduced to recalibrate the channels of $C_{1}$ and $C_{2}$, thereby emphasizing discriminative channels associated with *Ulva prolifera* boundaries.

After channel recalibration, the enhanced shallow features are forwarded simultaneously to two subsequent processing paths:

1. **Main decoding path.**
  This path retains the original SegFormer decoder and semantic segmentation head, ensuring that the overall segmentation framework remains unchanged.
2. **Boundary supervision branch (Boundary Branch).**
  This auxiliary branch utilizes the enhanced shallow features to learn boundary representations and provide additional supervision for contour prediction.

**Figure 2:**
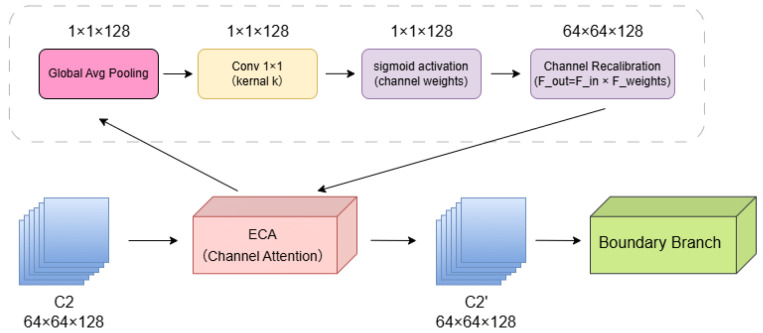
Structure of the ECA module.

The structure of the ECA module is illustrated in Figure 2.

Before entering both the decoder and the boundary branch, the shallow features are first processed by the ECA module for channel recalibration. Unlike the traditional SE attention mechanism, ECA avoids fully connected layers and channel dimensionality reduction. Instead, it employs a lightweight one-dimensional convolution to capture local cross-channel interactions, thereby improving feature discriminability while maintaining high computational efficiency.

The ECA module is applied to the outputs of $C_{1}$ and $C_{2}$ before they enter the decoder and the boundary branch, producing recalibrated features $C_{1}^{\prime }$ and $C_{2}^{\prime }$.

Specifically, let the input feature map be

$$F\in R^{H\times W\times C}$$

where *H* and *W* represent the spatial resolution and *C* denotes the number of channels. First, a global average pooling (GAP) is applied to obtain a channel descriptor vector:

$$\begin{array}{cc} z_{c} & =\frac{1}{H\times W}\sum_{i=1}^{H}\sum_{j=1}^{W}F_{i,j,c},\;z\in R^{C} \end{array}$$

This vector encodes the global response strength of each channel. ECA then applies a one-dimensional convolution with adaptive kernel size to model dependencies between adjacent channels:

$$\begin{array}{c} s\;=\;\sigma \left(\mathrm{Conv}1D_{k}\left(z\right)\right),\;s\in {\left[0,1\right]}^{C} \end{array}$$

where σ(·) denotes the Sigmoid function, and $s_{c}$ represents the importance weight of each channel.

Finally, the recalibrated feature map is obtained via channel-wise multiplication:

$${\hat{F}}_{i,j,c}\;=\;F_{i,j,c}\cdot s_{c}$$

The kernel size *k* is determined by an adaptive rule:

$$k\;=\;\mathrm{odd}\left(\left\lfloor \frac{\log_{2}\left(C\right)}{\gamma }+b\right\rfloor \right)$$

where γ and *b* are hyperparameters, set as γ=2, b=1 and odd(·) rounds to the nearest odd integer. For the shallow features in this study, $C_{1}$ has C=64, yielding k≈5; $C_{2}$ has C=128, also yielding k≈5. Therefore, a one-dimensional convolution with k=5 is used for both layers. This design significantly enhances the response of edge- and texture-related channels in shallow features with minimal computational overhead, providing clearer representations for both the boundary supervision branch and the decoder.

Here, $L_{\mathrm{seg}}$ denotes the semantic segmentation loss, $L_{\mathrm{bnd}}$ represents the boundary BCEWithLogitsLoss, and λ is a weighting factor. Since $C_{1}^{\prime }$ and $C_{2}^{\prime }$ are simultaneously fed into both branches, the gradients from both loss terms backpropagate to the shallow encoder features and the ECA weights. During training, this mechanism jointly shapes shallow features and channel weights, continuously reinforcing channels that are sensitive to *Ulva prolifera* boundaries.

This strategy not only enhances edge sensitivity in semantic segmentation and improves the purity of boundary prediction but also avoids additional computational overhead that would result from stacking complex attention modules within the boundary branch.

#### 3.2.2. Parallel Edge Supervision Branch to the Decoder

In remote sensing imagery, the boundaries of *Ulva prolifera* are often blurred, fragmented, or mixed with background noise. Since the optimization objective of the backbone and decoder networks is typically biased toward semantic consistency, the model tends to emphasize classification of the overall algal region while overlooking precise boundary localization. Consequently, conventional segmentation methods frequently produce discontinuous contours, jagged edges, or excessively smoothed boundaries, which ultimately affect the accuracy of area estimation.

**Figure 3:**
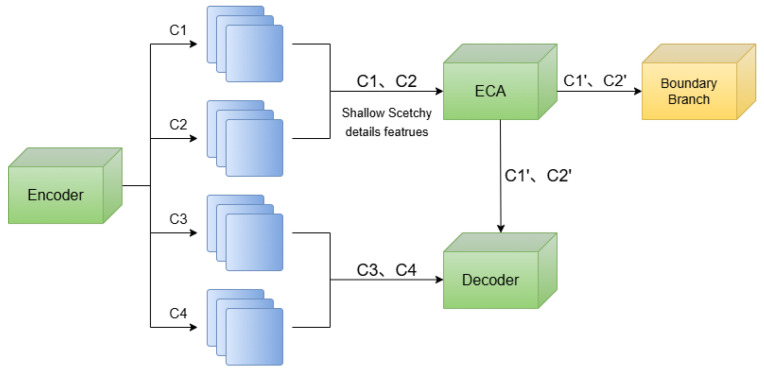
Location of the boundary supervision branch within the decoder module.

To address this issue, a **parallel boundary supervision branch** is incorporated into SegFormer (see Figure 3) to explicitly guide boundary learning during training and enhance boundary feature representation. The design of the boundary supervision branch mainly involves three aspects:

1. **Extraction of boundary-sensitive shallow features.**
  Shallow feature maps contain rich spatial and texture information that is beneficial for capturing subtle differences between foreground and background regions. These features are therefore utilized to extract boundary-sensitive representations and preserve fine-grained structural details.
2. **Boundary probability map generation.**
  Convolutional layers are applied to transform the extracted features into a boundary probability map, enabling the network to explicitly predict the contour distribution of *Ulva prolifera* regions.
3. **Edge-aware supervision.**
  Boundary supervision signals are generated using a morphological gradient operation applied to the binary ground-truth masks. Specifically, a square structuring element of size 3×3 is used for both dilation and erosion, and the boundary map is obtained as the difference between the dilated and eroded masks. The input masks are binarized without additional preprocessing. An edge-aware loss function is applied to constrain the predicted boundary map. This mechanism forces the network to explicitly learn the distribution of edge and non-edge pixels during training, thereby improving the continuity and accuracy of boundary extraction.

**Figure 4:**
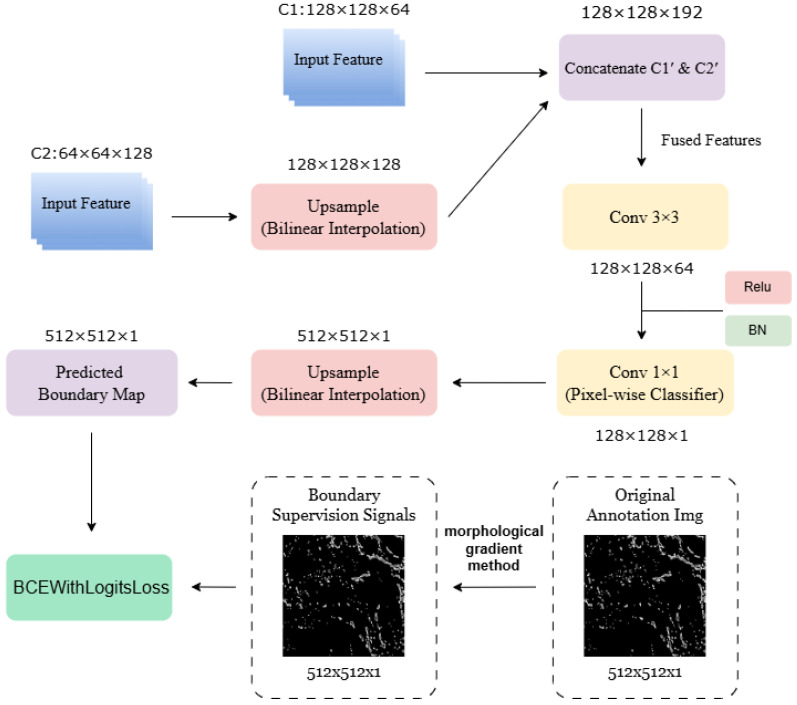
Structural design of the boundary supervision branch.

Unlike the main semantic segmentation head, which emphasizes regional class consistency, the boundary branch is specifically designed to differentiate edge from non-edge pixels, enforcing structural constraints during segmentation. Based on this principle, the structural design of the proposed boundary branch module is illustrated in Figure 4.

The operational workflow of the boundary branch is as follows:

1. **Input and feature alignment**
  The branch receives shallow features $C_{1}^{\prime }$ (size 128×128×64) and $C_{2}^{\prime }$ (size 64×64×128) from the encoder. To enable fusion, $C_{2}^{\prime }$ is upsampled to match the spatial dimensions of $C_{1}^{\prime }$ (128×128), and then the two feature maps are concatenated along the channel dimension, producing fused features of size 128×128×192.
2. **Local edge feature encoding**
  The fused features are processed with a 3×3 convolution to extract edge responses, followed by batch normalization (BN) to stabilize the feature distribution, and ReLU activation to suppress low-amplitude noise while preserving high-amplitude edge signals. Assuming the convolution output channels $F_{1}\;=\;64$, the resulting feature map has dimensions 128×128×64.
3. **Per-pixel boundary classification**
  A 1×1 convolution acts as a per-pixel linear classifier, integrating multi-source edge responses into a single-channel boundary logits map (128×128×1) representing the probability of each pixel belonging to a boundary.
4. **Boundary prediction upsampling**
  To align with the high-resolution boundary supervision signal (512×512×1), the predicted boundary logits map is upsampled using bilinear interpolation to 512×512×1, ensuring correct per-pixel loss computation.
5. **Edge-aware supervision and gradient feedback**
  A high-resolution boundary supervision signal is generated from the ground-truth mask using the same morphological gradient configuration described above and applied with BCEWithLogitsLoss to supervise the predicted map. Gradients propagate back to shallow features $C_{1}^{\prime }$ and $C_{2}^{\prime }$, and during decoder multi-scale fusion, indirectly enhance the main segmentation branch’s sensitivity to high-frequency edge information, thereby preserving structural details and improving boundary prediction accuracy.

During training, the parallel boundary supervision branch produces a predicted boundary map, which is upsampled to align with the resolution of the high-resolution ground-truth boundary signal. A binary cross-entropy loss with logits (BCEWithLogitsLoss) is employed to explicitly supervise the learning of edge and non-edge pixels. Gradients from the boundary branch are propagated back to the shallow encoder features $C_{1}^{\prime }$ and $C_{2}^{\prime }$, which are subsequently fused within the decoder. This supervision not only enforces explicit boundary constraints but also indirectly enhances the sensitivity of the main segmentation branch to high-frequency structural details. In other words, by guiding the shallow features with edge-aware supervision, the decoder can better preserve fine-grained contours during semantic segmentation, thereby effectively improving the accuracy and continuity of predicted boundaries.

Following this, we introduce the design of the loss function, which jointly optimizes the main segmentation and boundary branches to ensure balanced learning of semantic regions and precise boundaries.

The total loss is defined as

$$L_{\mathrm{total}}\;=\;L_{\mathrm{seg}}+\lambda L_{\mathrm{bnd}}$$

where $L_{\mathrm{seg}}$ denotes the semantic segmentation loss and $L_{\mathrm{bnd}}$ represents the boundary BCEWithLogitsLoss.

To investigate the effect of the boundary loss weighting factor λ on the segmentation performance, we conducted a detailed ablation study. The experiments were carried out on the HYU dataset using the full model, including the ECA module and the parallel boundary supervision branch. Specifically, we varied the boundary loss weight λ from 0 (effectively disabling boundary supervision) to 1.0, while keeping all other hyperparameters constant.

Each configuration was trained independently three times with different random seeds to assess the stability and statistical significance of the results. The evaluation metrics include the mean Intersection over Union (mIoU) for overall semantic segmentation accuracy and the boundary F1-score (BF-score) for boundary quality. The BF-score was computed using a tolerance distance of 3 pixels. Specifically, a predicted boundary pixel is regarded as correctly detected if it lies within 3 pixels of the ground-truth boundary, and vice versa. Boundary precision and recall are then calculated based on this matching criterion, and the BF-score is obtained as their harmonic mean, consistent with commonly adopted methods in boundary evaluation, ensuring that predicted edges within this distance from the ground-truth boundaries are considered true positives.

The results are summarized in Table 1, with all models maintaining a parameter count of 27.612 M, indicating that the observed performance differences primarily arise from adjustments to the boundary loss weight λ rather than model capacity. The results demonstrate that λ=0.5 achieves the optimal trade-off between semantic segmentation accuracy and boundary delineation quality. Smaller λ values weaken the boundary supervision effect, resulting in lower boundary fidelity, while larger λ values may slightly compromise segmentation performance in non-boundary regions. The standard deviations reported in the table show that this trend is consistent across three independent runs, indicating statistical reliability. Overall, this ablation study confirms the effectiveness of the boundary supervision branch in improving boundary accuracy. The final selection of λ=0.5 ensures stable and significant improvements in both segmentation performance and boundary quality, with repeated experiments further validating the robustness of this setting.

### 3.3. Network Complexity and Parameter Analysis

The proposed modifications—namely the Efficient Channel Attention (ECA) module and the boundary supervision branch—introduce only a minimal number of additional parameters to the original SegFormer architecture. From a principled perspective, this can be explained as follows:

1. **Efficient Channel Attention (ECA) module**
  The ECA module recalibrates channel-wise feature responses by employing a lightweight one-dimensional convolution, entirely avoiding fully connected layers or explicit channel dimensionality reduction. The convolution kernel size is small (typically 3×5 or 5), and the operation is applied only to shallow feature maps $C_{1}$ and $C_{2}$, which have relatively low channel dimensions (64 and 128 channels, respectively). Consequently, the number of additional trainable parameters introduced by ECA is extremely limited. Despite this minimal parameter overhead, the module effectively enhances inter-channel feature interactions, improving the representational capacity of shallow features without altering the overall network structure.
2. **Boundary supervision branch**
  The boundary branch is designed to generate auxiliary boundary probability maps from the recalibrated shallow features. It consists of a 3×3 convolution followed by batch normalization (BN) and ReLU activation, and a subsequent 1×1 convolution that outputs a single-channel boundary map. Since both the input channels and the output channel are small, the total number of trainable weights in this branch is very low. Additionally, the branch is used solely for supervision during training and does not participate in the main decoder’s forward inference. As a result, it imposes negligible additional computational or memory burden while providing effective guidance for learning high-frequency edge information.

The model parameters and computational complexity (GFLOPs) were computed using the thop library in *PyTorch 1.13* with *CUDA 11.7*. The total number of parameters corresponds to all learnable weights in the network, while GFLOPs were estimated based on the number of multiply-accumulate operations (MACs) during a single forward pass with an input resolution of 512×512. All computations were performed under a consistent experimental setting to ensure fair comparison across different models. The scripts used for these calculations can be provided upon request for reproducibility.

In summary, both the ECA module and the boundary supervision branch are lightweight and computationally efficient. The ECA module improves inter-channel feature representation in shallow layers, and the boundary branch provides high-resolution edge guidance during training. Taken together, these improvements result in only a minor increase in the total number of parameters, consistent with the parameter counts reported in Table 2. This demonstrates that the proposed modifications achieve enhanced feature representation and boundary learning without substantially increasing model complexity or computational cost.

## 4. Experiments and Analysis

### 4.1. Experimental Dataset

The remote sensing data used in this study are derived from China’s independently developed **Coastal Zone Imager (CZI, 50 m resolution)** onboard the HY-1C, HY-1D, and HY-1E satellites. The HY-1C satellite, launched in 2018, is equipped with both the Coastal Zone Imager (CZI) and the **Medium-Resolution Imaging Spectrometer (PMRIS, 1 km resolution)**, enabling large-scale and continuous observations of ocean color parameters. The HY-1D satellite, launched in 2020, provides improved spectral resolution and radiometric accuracy, while HY-1E further enhances spatiotemporal resolution and quantitative observation capability. These satellites cover key nearshore regions of China, especially the Yellow Sea, offering reliable data support for long-term monitoring of *Ulva prolifera* blooms.

Compared with widely used international satellites such as Landsat, Sentinel-2, and MODIS, the HY-1 series is specifically designed for ocean color observation and exhibits strong sensitivity to water spectral characteristics. This makes it particularly suitable for detecting marine phenomena such as *Ulva prolifera* green tides.

The dataset used in this study, referred to as the **HYU dataset**, consists of remote sensing images in **GeoTIFF format** along with corresponding *Ulva prolifera* mask annotations. The dataset used in this study, referred to as the **HYU dataset**, consists of remote sensing images in **GeoTIFF format** along with corresponding *Ulva prolifera* mask annotations. The original data contain multiple spectral bands, from which a subset of bands is selected for model input. Detailed input configuration and preprocessing strategies are described in Section 4.3.

Ground-truth annotations are generated from vector boundaries obtained through inversion and expert interpretation. These vector files (shapefiles) are subsequently rasterized to produce pixel-wise mask labels aligned with the input images.

The original data contain multiple spectral bands, from which a subset of bands is selected for model input. In this study, a near-infrared (NIR) together with blue-green (BG) band combination is adopted instead of the conventional red-based configuration.

This choice is consistent with widely adopted practices in marine and aquatic remote sensing, where NIR-based band combinations are commonly used to enhance the contrast between floating targets and surrounding water bodies [26,58,59,60].

Specifically, due to the strong absorption of NIR radiation by water, the background typically exhibits very low reflectance, while floating algae show relatively higher reflectance, resulting in a more distinguishable spectral response. Meanwhile, the blue-green bands are more sensitive to variations in water constituents such as chlorophyll concentration, suspended matter, and shallow-water features, providing complementary information for discriminating *Ulva prolifera* from complex marine backgrounds [61,62,63].

The red band is highly sensitive to water turbidity and suspended particles [64,65,66], which may introduce background interference in marine environments. In this study, the target region is the Yellow Sea and Bohai Sea, which are characterized by relatively high turbidity and abundant suspended sediments [67,68,69]. Under such conditions, incorporating the red band is more likely to amplify turbidity-related signals and introduce additional interference, thereby reducing its discriminative capability for *Ulva prolifera*. As a result, its effectiveness becomes limited, especially under complex conditions with varying turbidity levels. In contrast, the near-infrared (NIR) band provides more stable and distinctive responses, as water exhibits strong absorption while floating algae show high reflectance. Therefore, replacing the red band with NIR can effectively enhance the separability between *Ulva prolifera* and surrounding water.

Therefore, the NIR-BG combination provides improved discriminability and robustness for *Ulva prolifera* detection under complex coastal conditions.

**Figure 5:**
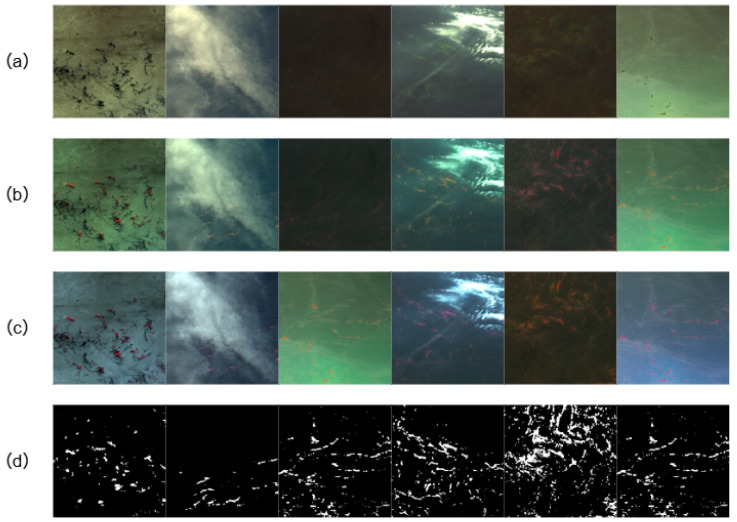
Examples of the HYU dataset: (**a**) RGB composite (channels 3, 2, 1), (**b**) false-color composite (channels 4, 3, 2), (**c**) NIR-based composite used in this study (channels 4, 2, 1), (**d**) corresponding mask annotation.

Representative samples of the dataset are shown in Figure 5.

The dataset is partitioned as shown in Table 3.

### 4.2. Evaluation Metrics

To validate the effectiveness of the proposed improvements, ablation experiments were conducted on the HYU dataset using SegFormer as the baseline system. Model performance was evaluated using mean Intersection over Union (mIoU), F1 scores, precision, and recall.

**mIoU** measures the average percentage of overlap between predicted and actual *Ulva prolifera* pixels.

**The F1 score** considers both precision and recall, providing a balanced measure of model accuracy.

**Precision** represents the proportion of correctly predicted *Ulva prolifera* pixels among all predicted *Ulva prolifera* pixels.

**Recall** denotes the proportion of correctly predicted *Ulva prolifera* pixels among all actual *Ulva prolifera* pixels.

Let *T* and *F* denote predicted Ulva and non-Ulva pixels, respectively, and *P* and *N* denote actual Ulva and non-Ulva pixels, respectively. The formulas for the four evaluation metrics are as follows:

$$\begin{array}{cc} \mathrm{mIoU} & =\frac{1}{K+1}\sum_{i=0}^{K}\frac{TP_{i}}{TP_{i}+FP_{i}+FN_{i}}\\ F1 & =\frac{2\cdot \mathrm{Precision}\cdot \mathrm{Recall}}{\mathrm{Precision}+\mathrm{Recall}}\\ \mathrm{Precision} & =\frac{TP}{TP+FP}\\ \mathrm{Recall} & =\frac{TP}{TP+FN} \end{array}$$

To further evaluate the effectiveness of the proposed boundary supervision branch, boundary-aware evaluation metrics are introduced in addition to region-based metrics such as mIoU and F1-score. Given that the proposed method explicitly enhances boundary representation, these metrics provide a more targeted assessment of contour quality.

Specifically, the Boundary F1-score (BF-score) is employed to measure the alignment between predicted boundaries and ground-truth contours. Let $B_{p}$ and $B_{g}$ denote the predicted and ground-truth boundary sets, respectively. A predicted boundary pixel is considered a true positive if there exists at least one ground-truth boundary pixel within a tolerance distance δ from it, and vice versa for ground-truth boundary pixels. In this study, δ is set to 3 pixels, following common practice in boundary evaluation.

The precision and recall of boundary extraction are defined as:

$$\begin{array}{c} {\mathrm{Precision}}_{b}\;=\;\frac{|B_{p}\cap B_{g}^{\delta }|}{|B_{p}|}\\ {\mathrm{Recall}}_{b}\;=\;\frac{|B_{g}\cap B_{p}^{\delta }|}{|B_{g}|} \end{array}$$

where $B_{g}^{\delta }$ denotes the δ-neighborhood of the ground-truth boundary, and $B_{p}^{\delta }$ is defined analogously.

The BF-score is then computed as:

$$\begin{array}{c} \mathrm{BF-score}\;=\;\frac{2\cdot {\mathrm{Precision}}_{b}\cdot {\mathrm{Recall}}_{b}}{{\mathrm{Precision}}_{b}+{\mathrm{Recall}}_{b}} \end{array}$$

In addition, the Hausdorff Distance (HD) is adopted to evaluate the maximum deviation between predicted and ground-truth boundaries, providing a stricter assessment of contour accuracy:

$$\begin{array}{c} \mathrm{HD}\left(B_{p},B_{g}\right)\;=\;\max\left\{\underset{x\in B_{p}}{\sup}\underset{y\in B_{g}}{\inf}d\left(x,y\right),\;\underset{y\in B_{g}}{\sup}\underset{x\in B_{p}}{\inf}d\left(x,y\right)\right\} \end{array}$$

where d(x,y) denotes the Euclidean distance between two boundary points. A smaller HD indicates better boundary alignment.

Overall, these boundary-focused metrics complement traditional region-based metrics, enabling a more comprehensive and reliable evaluation of segmentation performance, particularly for boundary-sensitive tasks such as *Ulva prolifera* semantic segmentation. 

### 4.3. Experimental Settings

#### 4.3.1. Experimental Environment and Training Settings

All experiments in this study were conducted on a server equipped with an **NVIDIA GeForce RTX 4090 GPU**. The operating system is **Ubuntu 20.04**, and the deep learning framework used is **PyTorch 1.13** with **CUDA 11.7**. Data preprocessing and visualization were performed using **Python 3.8** along with common scientific computing libraries such as **NumPy 1.23.5**, **OpenCV 4.6.0**, and **Matplotlib 3.5.3**.

The training configurations of the dataset are summarized in Table 4.

#### 4.3.2. Input Configuration and Preprocessing

The original data used in this study are multispectral remote sensing images in GeoTIFF format, containing multiple spectral bands. During the preprocessing stage, three bands, namely near-infrared (NIR), green (G), and blue (B), are selected from the original data. The NIR band is used to replace the conventional red (R) band, forming a three-channel (NIR, G, B) composite, which enhances the spectral separability between Ulva prolifera and the surrounding seawater.

Considering the large spatial coverage and high resolution of remote sensing images, a sliding-window strategy is employed to crop the original images into patches. Specifically, image tiles of size 512×512 are generated to serve as inputs for model training. During this process, vector data representing ocean regions are utilized to filter the generated patches, retaining tiles that intersect with target areas as well as a small number of background ocean samples, while excluding irrelevant regions such as land and dense clouds, thereby improving data utilization efficiency.

For spectral preprocessing, a global percentile-based contrast stretching method is adopted to reduce radiometric differences among images and enhance contrast. Specifically, the 2nd and 98th percentiles are computed for each band, followed by linear stretching to normalize pixel values into the range of [0,1]. The values are then scaled to [0,255] and converted to 8-bit integers for generating PNG images.

For annotation generation, vector boundaries obtained from manual interpretation are rasterized to produce pixel-wise mask labels that are spatially aligned with the image tiles. All preprocessing operations are consistently applied across the training, validation, and test sets to ensure fairness and reproducibility of the experiments.

This preprocessing pipeline ensures spectral consistency while improving data processing efficiency, making it suitable for large-scale remote sensing semantic segmentation tasks.

#### 4.3.3. Data Augmentation

After input configuration and preprocessing, online data augmentation is applied to the generated PNG image patches during training to improve the generalization ability of the model and alleviate overfitting. Considering the large-scale variations, diverse observation angles, and complex sea surface textures in remote sensing imagery, a combination of geometric and spectral augmentation strategies is adopted.

For geometric augmentation, random horizontal flipping and vertical flipping are independently applied with a probability of 0.5 to enhance robustness to different viewing directions. In addition, random rotations (i.e., $90^{{}^{\circ}}$, $180^{{}^{\circ}}$, and $270^{{}^{\circ}}$) are introduced to further improve the model’s adaptability to orientation variations. Meanwhile, under the constraint of a fixed input size of 512×512, random cropping and scale perturbation are employed to simulate observations at different spatial resolutions.

For spectral augmentation, controlled perturbations in brightness and contrast are applied to simulate varying imaging conditions and marine environments. Considering that the color differences between small-scale Ulva prolifera targets and the background, as well as those between large-scale boundaries and seawater, are relatively subtle, saturation variation is not introduced to avoid altering discriminative spectral characteristics.

Specifically, the magnitudes of brightness and contrast perturbations are constrained within a limited range (±10%), ensuring that the augmentation only induces minor adjustments to the overall radiometric distribution without changing the relative spectral relationships between Ulva prolifera and the background. This strategy mitigates the influence of illumination changes, atmospheric variations, and sensor inconsistencies while preserving semantic consistency, and complements the proposed boundary supervision branch, thereby improving segmentation accuracy in boundary regions under complex marine backgrounds.

It should be noted that all data augmentation operations are only applied during the training phase, while no augmentation is performed on the validation and test sets to ensure objective and fair evaluation.

This augmentation strategy effectively expands the training data distribution without altering label consistency, thereby improving the model’s adaptability to complex marine environments.

### 4.4. Ablation Experiment

#### 4.4.1. Ablation Experiments on Modules with Different Mechanisms

The original SegFormer model and various network variants incorporating different mechanisms were evaluated on the HYU dataset. The comparison of evaluation metrics is summarized in Table 5.

From the ablation results, it can be observed that introducing either the boundary supervision branch or the ECA channel attention module alone improves segmentation performance. Specifically, the boundary supervision branch notably enhances **recall** (from 69.84% to 75.67%) and the BFScore (from 77.91% to 83.06%), demonstrating its effectiveness in boundary localization and fine-grained structure recovery. Meanwhile, embedding the ECA module into shallow features improves **precision** (from 91.75% to 92.84%) and the BFScore (from 77.91% to 80.62%), indicating that channel attention strengthens feature discrimination while suppressing irrelevant responses. The Hausdorff Distance (HD) also decreases (from 17.42 to 15.03 pixels), reflecting improved boundary continuity.

When both mechanisms are combined in the proposed network, all metrics achieve the best performance. Compared with the baseline SegFormer, **mIoU, F1, precision, and recall** improve by 6.80%, 4.76%, 2.37%, and 6.22%, respectively, while the **BFScore** increases by 6.45%, and **HD** decreases by 5.95 pixels. These results demonstrate that integrating boundary-aware learning and channel attention not only complements the encoder–decoder backbone but also simultaneously enhances global semantic consistency and local boundary accuracy, leading to substantial overall improvements in segmentation performance.

**Figure 6:**
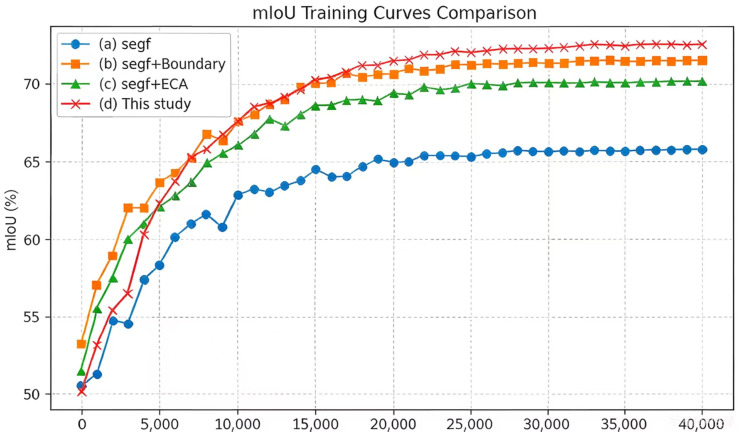
mIoU curves during training for the baseline and improved networks.

**Figure 7:**
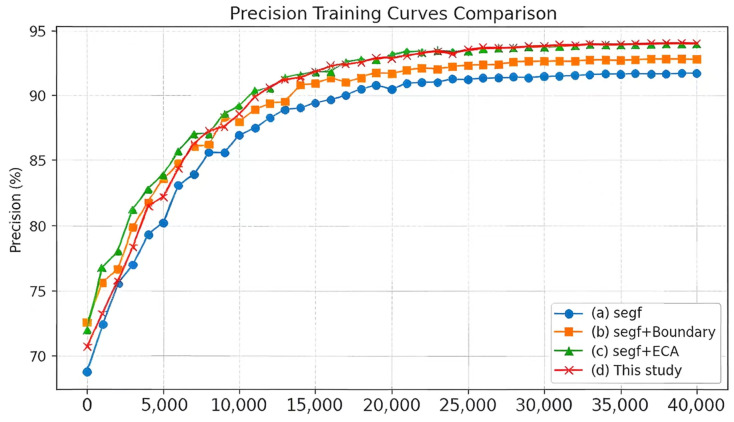
Precision curves during training for the baseline and improved networks.

The changes in segmentation accuracy during training for the original and improved SegFormer models are shown in Figure 6 and Figure 7.

#### 4.4.2. Ablation Experiments on the Embedding Layer of the ECA Module

To further investigate the effect of the ECA module at different network layers, multiple ablation experiments (A1-D2) were designed. They were conducted to determine the optimal insertion position of the ECA module. The shallow layers correspond to the first two encoder layers [0, 1], and the deep layers correspond to the last two layers [2, 3]. These configurations were evaluated both independently and in combination with the boundary supervision branch. The comparison of evaluation metrics is summarized in Table 6.

Experimental results are summarized in Table 6. Without boundary supervision, activating ECA in shallow layers (A1) mainly improved **g** (74.14%) compared with the baseline, confirming that shallow channel attention strengthens responses to edges and texture details. However, due to limited suppression of false positives, the gain in **precision** was modest (93.01%). Activating ECA in deeper layers (A2) yielded a more substantial improvement in **precision** (94.49%) but a relatively smaller increase in recall (72.83%), indicating that high-level semantic attention better suppresses background interference and enhances class discrimination.

After introducing boundary supervision, shallow-layer ECA with boundary supervision (B1) significantly boosted **recall** (75.67%) and the **BFScore** (82.75%), enabling more accurate boundary and fine-structure recovery. Deep-layer ECA with boundary supervision (B2) further improved **precision** (93.90%) and overall consistency (**mIoU = 71.88%**, **F1 = 83.64%**, **HD = 15.12**).

When combining ECA with boundary supervision at specific feature layers, the proposed network (D2) achieved the best performance across all metrics: **mIoU = 72.61%**, **F1 = 84.14%**, **Precision = 94.12%**, **Recall = 76.06%**, **BFScore = 84.36%**, and **HD = 11.47 pixels**. This demonstrates that activating ECA in shallow layers under boundary supervision effectively balances global semantic modeling and local boundary detail recovery, substantially enhancing segmentation performance.

Overall, the results indicate that shallow-layer ECA is particularly effective when combined with boundary supervision, as it directly strengthens low-level spatial details crucial for precise boundary delineation.

### 4.5. Comparative Experiments

#### 4.5.1. Baseline Model Comparative Experiments

In this experiment, the prediction results of the proposed method were compared with the ground-truth labels and several representative semantic segmentation networks to evaluate its effectiveness. The selected baseline models include **HRNet**, **PSPNet**, **U-Net**, the original **SegFormer**, and **SegFormer-ASPP** (SegFormer with Atrous Spatial Pyramid Pooling). The quantitative comparison results are summarized in Table 7.

The proposed method outperforms traditional convolutional networks (HRNet, PSPNet, and U-Net) as well as the original SegFormer model on the HYU dataset. Specifically, compared with the baseline SegFormer, the proposed method improves **mIoU** from **65.81%** to **72.61%**, **F1** from **79.38%** to **84.14%**, **Precision** from **91.75%** to **94.12%**, and **Recall** from **69.84%** to **76.06%**. Meanwhile, the **BFScore** increases from **77.91%** to **84.36%**, and the **Hausdorff Distance (HD)** decreases from **17.42** pixels to **11.47** pixels, indicating a substantial improvement in both regional segmentation accuracy and boundary localization quality. The mIoU gain reaches up to **10.32%** compared with the best baseline (SegFormer), while the BFScore improvement reaches **8.31%**, further highlighting the effectiveness of the proposed strategy in jointly enhancing semantic consistency and boundary detail recovery.

From the comparative results, U-Net and PSPNet exhibit limitations in recovering fine boundary structures and suppressing complex marine background interference, resulting in relatively lower F1, Recall, and BFScore values, as well as larger HD values. In contrast, the proposed method leverages shallow-layer ECA enhancement and boundary supervision to improve edge-sensitive feature responses while preserving global semantic information, thereby achieving simultaneous improvements in precision, recall, and boundary continuity.

In addition, SegFormer-ASPP (SegF-ASPP) performs slightly worse than the original SegFormer, with **mIoU** and **Recall** decreasing by **1.49%** and **2.23%**, respectively. Its BFScore is also slightly lower, while the HD value increases, suggesting weaker boundary consistency. This indicates that although ASPP improves multi-scale context aggregation, it may weaken the representation of small fragmented targets and boundary continuity in this task. These results further demonstrate that, for *Ulva prolifera* with irregular morphology and weak boundaries, shallow feature enhancement and explicit boundary learning are more effective optimization strategies.

Overall, the proposed network significantly improves semantic segmentation accuracy, particularly in complex boundary extraction, small-object segmentation, and robustness to marine noise. The consistent improvements in both BFScore and HD further verify the superiority of the proposed method in boundary-aware segmentation.

The model complexity and efficiency of HRNet, PSPNet, U-Net, the original SegFormer, and SegFormer-ASPP were compared with the proposed method, as summarized in Table 8.

In this experiment, GFLOPs are computed with an input size of 512 × 512. The computation time refers to the average time required to complete a single iteration (i.e., one forward and backward pass of a batch) on the HYU dataset.All computation times are measured under the same experimental conditions, including identical hardware (NVIDIA RTX 4090), batch size (8), and input resolution (512 × 512). The reported time corresponds to the average iteration time over multiple batches after a warm-up phase, excluding data loading overhead. This ensures a fair comparison of computational efficiency across different models.

The proposed method introduces lightweight improvements to SegFormer, including the ECA module and the boundary supervision branch. As a result, the number of parameters increases only slightly from 27.5 M to 27.612 M, while the computational cost remains comparable. Despite this negligible increase in model complexity, a significant improvement in mIoU is achieved, demonstrating a favorable trade-off between computational efficiency and segmentation performance, and indicating strong practical applicability.

**Figure 8:**
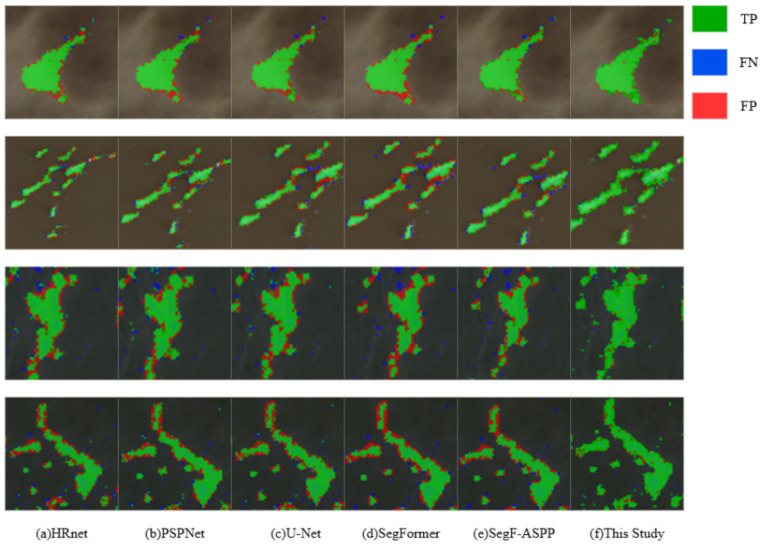
Comparison of semantic segmentation results across different networks.

Figure 8 presents the prediction results of HRNet, PSPNet, U-Net, the original SegFormer, SegFormer-ASPP, and the proposed method on the HYU dataset. Correctly predicted pixels are marked in **green**, false negatives in **blue**, and false positives in **red**. Visual inspection highlights several advantages of the proposed network over other methods:

1. **Superior boundary delineation and smoother edge transitions**
  As shown in Figure 8, the proposed network produces more continuous and accurate boundary contours, particularly in low-contrast regions where Ulva patches merge with complex marine backgrounds. Compared with other methods, the predicted boundaries are smoother and more consistent with the ground truth.
2. **Improved segmentation of small and heterogeneous patches**
  The proposed method demonstrates stronger capability in recovering small scattered Ulva patches and heterogeneous mixed regions. Benefiting from shallow-layer ECA enhancement and explicit boundary supervision, the model effectively reduces missed predictions and preserves clearer local structures.
3. **Better detail preservation in complex aggregation regions**
  In regions containing multiple adjacent patches, the proposed network preserves finer edge details and reduces over-smoothing effects, resulting in segmentation contours that more closely align with manual annotations.
4. **Remaining challenges under severe environmental interference**
  Despite the improved boundary sensitivity and semantic discrimination, minor false positives and false negatives may still occur under strong reflections, water ripples, or surrounding plankton interference, suggesting room for further optimization in complex spectral environments.

#### 4.5.2. Comparison with Representative Advanced Segmentation Models

To further validate the effectiveness, robustness, and generalization capability of the proposed method, additional comparative experiments were conducted using several representative advanced semantic segmentation models on the HYU dataset. The selected baselines include **DeepLabV3+**, **Swin-UNet**, **Gated-SCNN**, and **OCRNet**, which respectively represent strong convolutional neural network-based architectures, recent Transformer-based segmentation frameworks, classical boundary-aware segmentation methods, and high-performance context modeling networks.

The selection of these comparison models is based on their wide adoption, strong citation impact, and representative methodological characteristics in the field of semantic segmentation. Specifically, DeepLabV3+ is introduced as a widely recognized CNN-based baseline with excellent multi-scale contextual modeling capability. Swin-UNet is employed as a representative hierarchical Transformer segmentation architecture with strong global feature modeling ability. Gated-SCNN is selected as a classical boundary-aware segmentation framework to evaluate the effectiveness of the proposed boundary supervision strategy. OCRNet is further included as a strong semantic segmentation baseline with enhanced object-context representation capability.

By comparing the proposed method with these representative models, this study aims to comprehensively evaluate its performance in terms of global semantic consistency, local boundary delineation, and robustness under complex marine background interference.

As shown in Table 9, the proposed method consistently outperforms both classical convolutional networks and recent advanced segmentation architectures on the HYU dataset. Specifically, compared with the strongest baseline OCRNet, the proposed method further improves **mIoU** from 71.88% to 72.61%, **F1-score** from 83.75% to 84.14%, **Precision** from 93.67% to 94.12%, and **Recall** from 75.58% to 76.06%. Although the absolute gains appear moderate, such improvements are highly meaningful given the already strong performance of the compared advanced models.

Compared with the CNN-based baseline DeepLabV3+, the proposed method improves mIoU by 2.77% and the BFScore by 5.80%, indicating that the proposed architecture achieves better global semantic consistency while substantially enhancing boundary delineation capability. This improvement mainly benefits from the complementary design of shallow-layer ECA feature enhancement and explicit boundary supervision, which is more suitable for irregular floating *Ulva prolifera* contours than purely context-driven CNN architectures.

Compared with the Transformer-based Swin-UNet, the proposed method achieves higher performance across all region-based and boundary-based metrics. In particular, the BFScore increases from 80.42% to 84.36%, while HD decreases from 14.28 to 11.47 pixels. This demonstrates that, although hierarchical Transformer architectures possess strong global modeling ability, they may still be insufficient in capturing weak boundaries and small scattered patches under low-contrast marine conditions. In contrast, the proposed boundary supervision branch explicitly constrains contour learning, significantly improving local structural recovery.

More importantly, compared with the representative boundary-aware model Gated-SCNN, the proposed method still achieves superior results in both semantic and boundary metrics, with mIoU increasing by 1.25%, the BFScore improving by 1.63%, and HD decreasing by 1.44 pixels. This result strongly demonstrates that the proposed boundary supervision branch is not a simple auxiliary edge branch, but a task-oriented optimization strategy specifically designed for the complex boundary characteristics of *Ulva prolifera* in marine remote sensing imagery.

From a mechanism perspective, the shallow-layer ECA modules enhance edge-sensitive channel responses and improve discrimination between *Ulva prolifera* and surrounding seawater, particularly in low-contrast regions and small fragmented patches. Meanwhile, the boundary supervision branch provides explicit contour constraints, effectively reducing false positives caused by wave reflections and false negatives caused by weak algae-water transitions. The combination of these two components enables the network to simultaneously preserve global semantic consistency and local boundary continuity. Overall, the quantitative results confirm that the proposed method achieves a better balance between semantic representation, boundary sensitivity, and robustness to complex marine interference, thereby providing a more reliable solution for large-scale *Ulva prolifera* monitoring.

These results further demonstrate that the proposed method is not merely a simple boundary-branch extension, but a task-oriented optimization framework specifically designed for marine *Ulva prolifera* semantic segmentation. 

## 5. Conclusions

This study presents ECAB-SegFormer, an enhanced SegFormer-based network for high-precision semantic segmentation of *Ulva prolifera* in remote sensing imagery. The network integrates a boundary supervision branch and embeds Efficient Channel Attention (ECA) modules in shallow decoder layers to improve edge-sensitive feature representation while retaining the original SegFormer encoder–decoder structure. This design effectively addresses challenges caused by irregular Ulva morphology, small scattered patches, and complex marine backgrounds.

Extensive experiments on the HYU dataset demonstrate that ECAB-SegFormer consistently outperforms classical convolutional networks (HRNet, PSPNet, and U-Net) and recent segmentation models (SegFormer, SegFormer-ASPP, Swin-Unet, DeeplabV3+, and Gated-SCNN). Specifically, compared with the original SegFormer, ECAB-SegFormer improves **mIoU** from 65.81% to 72.61%, **F1** from 79.38% to 84.14%, **Precision** from 91.75% to 94.12%, **Recall** from 69.84% to 76.06%, and the **BFScore** from 81.46% to 86.34% and reduces the **Hausdorff Distance (HD)** from 12.85 to 9.35. These improvements highlight the network’s ability to capture fine boundaries, accurately segment small or heterogeneous Ulva patches, and suppress false positives and false negatives under complex marine conditions.

The results confirm that the proposed method achieves superior edge delineation, robust segmentation in challenging marine environments, and strong generalization across varied spatial distributions. The main contributions of this work include: (1) embedding ECA modules in shallow decoder layers to enhance feature discrimination, (2) introducing a boundary supervision branch to explicitly model and preserve edges, and (3) demonstrating state-of-the-art performance over both classical CNNs and modern Transformer-based segmentation networks. ECAB-SegFormer provides a reliable tool for large-scale Ulva monitoring, ecological early warning, and intelligent marine mapping, with future work focused on further improving robustness against spectral imaging interference. 

## Figures and Tables

**Table 1:** Ablation study on boundary loss weight λ with three independent runs.

λ	mIoU (%)	Std (%)	BF-Score (%)	Std (%)
0 (only ECA)	70.20	0.15	80.54	0.42
0.1	72.10	0.12	82.63	0.35
0.3	72.43	0.10	83.95	0.30
**0.5**	**72.61**	0.11	**84.36**	0.29
0.7	72.05	0.13	84.10	0.31
1.0	71.80	0.14	83.72	0.32

**Table 2:** Parameter allocation of the proposed network.

Module	Description	Parameters (M)
Encoder (SegFormer)	Original SegFormer hierarchical Transformer encoder	21.5
Decoder (SegFormer)	Original MLP decoder	1.9
Segmentation Head	Original final segmentation classifier	3.1
ECA Module	Efficient Channel Attention on shallow features (C1, C2)	0.05
Boundary Supervision Branch	3 × 3 Conv + BN + ReLU → 1 × 1 Conv for boundary prediction	0.06
**Total**		**27.612**

**Table 3:** Dataset split of the HYU dataset.

Dataset Partition	Number of Images	Size
train	1559	512 × 512
val	437	512 × 512
test	229	512 × 512

**Table 4:** Training configurations of the HYU dataset.

Item	Value
Input size	512×512
Train size	512 × 512
Test size	512×512
Iter	40,000
Batch size	8
Optimizer	AdamW
Learning decay	Linear Warmup + Poly
Weight decay	0.01
Learning rate	$6\times 10^{-5}$

**Table 5:** Ablation study: Comparison of segmentation performance between the original network and improved variants on the HYU dataset.

Network	mIoU (%)	F1 (%)	Precision (%)	Recall (%)	BFScore (%)	HD (Pixel)
(a) SegFormer	65.81	79.38	91.75	69.84	77.91	17.42
(b) SegF + Boundary	71.57	83.02	92.05	75.67	83.06	12.58
(c) SegF + ECA(C1-C2)	70.20	80.55	92.84	72.65	80.62	15.03
(d) Proposed (This study)	72.61 ↑6.80	84.14↑4.76	94.12↑2.37	76.06↑6.22	84.36↑6.45	11.47↓5.95

↑ and ↓ indicate performance improvement and reduction relative to the baseline (SegFormer), respectively.

**Table 6:** Ablation study on the HYU dataset with boundary and ECA modules.

Group	Activated Layer	Boundary Branch	mIoU (%)	F1 (%)	Precision (%)	Recall (%)	BFScore (%)	HD (Pixel)
A1	C1	X	69.02	81.67	93.01	74.14	78.01	16.83
A2	C2	X	70.94	82.98	94.49	72.83	79.62	15.33
B1	C3	✓	71.50	83.38	92.85	75.67	82.75	14.28
B2	C4	✓	71.88	83.64	93.90	75.42	83.06	15.12
D1	C3, C4	✓	70.32	82.58	92.34	74.68	82.95	13.97
D2 (Proposed)	C1, C2	✓	72.61	84.14	94.12	76.06	84.36	11.47

**Table 7:** Comparison of segmentation performance of different methods on the HYU dataset.

Evaluation Indicator	HRNet	PSPNet	U-Net	SegFormer	SegF-ASPP	Methods of This Paper	Best Improvement (%)
mIoU (%)	63.42	60.37	58.27	65.81	64.32	72.61	**10.32**
F1 (%)	77.14	75.03	73.62	79.38	76.02	84.14	**5.96**
Precision (%)	90.08	89.47	88.96	91.75	92.49	94.12	**1.77**
Recall (%)	68.12	65.42	62.85	69.84	67.61	76.06	**8.94**
BFScore (%)	74.63	71.28	69.84	77.91	76.35	84.36	**8.31**
HD (pixel) ↓	19.84	22.31	24.67	17.42	18.15	11.47	**36.82**

↓ indicates that lower values are better.

**Table 8:** Analysis of network complexity and efficiency.

Network	GFLOPS/G	Parameters/M	Computation Time (Sec/Iteration)	mIoU (%)
HRNet	50.3	65.1	5.67	63.42
PSPNet	45.6	48.7	3.85	60.37
U-Net	25.4	31.2	1.37	58.27
SegFormer	21.0	27.5	1.21	65.81
SegF-ASPP	23.5	30.2	1.35	64.32
Method of this paper	29.5	27.612	1.63	72.61

**Table 9:** Comparison with representative advanced segmentation models on the HYU dataset.

Method	mIoU (%)	F1 (%)	Precision (%)	Recall (%)	BFScore (%)	HD (Pixel)
DeepLabV3+	69.84	82.21	92.37	74.08	78.56	15.73
Swin-UNet	70.95	83.04	93.15	74.89	80.42	14.28
Gated-SCNN	71.36	83.37	93.02	75.46	82.73	12.91
OCRNet	71.88	83.75	93.67	75.58	81.95	13.24
**Proposed**	**72.61**	**84.14**	**94.12**	**76.06**	**84.36**	**11.47**

## Data Availability

Restrictions apply to the availability of these data. The datasets used in this study were obtained from the National Satellite Ocean Application Service (NSOAS) and are not publicly available due to data usage restrictions. Data may be available from the corresponding author upon reasonable request and with permission from the data provider.
